# Discourse2Draft: an AI-powered structured document writing tool

**DOI:** 10.3389/frai.2026.1945655

**Published:** 2026-10-27

**Authors:** Amlan Talukder, Parker K. Combs, Charles P. Schmitt, David M. Reif, Scott S. Auerbach

**Affiliations:** 1 Office of Data Science (ODS), National Institutes of Health (NIH)/National Institute of Environmental Health Sciences (NIEHS), Durham, NC, United States; 2 22nd Century Technologies, McLean, VA, United States; 3 Predictive Toxicology Branch (PTB), Division of Translational Toxicology (DTT), National Institutes of Health (NIH)/National Institute of Environmental Health Sciences (NIEHS), Durham, NC, United States; 4 Axle Informatics, Rockville, MD, United States

**Keywords:** AI, AI workflow, deep research, document writing, LLM agents, RAG

## Abstract

**Introduction:**

We introduce Discourse2Draft, a model-agnostic, AI-powered, advanced Retrieval-Augmented Generation (RAG) based application that converts a user-provided query on a topic into a polished full-form draft written in user-provided outline, using context from user-provided documents or literature downloaded from PubMed or both. This application can be used to generate any document that needs a structured outline.

**Methods:**

Discourse2Draft can take the outline of a document directly from the user or generate one from the user-provided query using AI. With a structured outline (AI-generated or user-provided) at its disposal, it first extracts the hierarchical structure from the outline and detects positions to write content. It then uses AI to extract core ideas from the corresponding section headers and previously written text for every writable position and to fill those positions with cohesive prose with context taken from user-provided documents or literature. During content generation, it automatically weaves source-anchored citations (user-provided documents or literature) throughout the text. The generated draft with associated references and the bibliography can be exported in Microsoft Word, LaTeX and Markdown formats, ensuring full transparency and downstream editability.

**Results:**

Discourse2Draft achieved competitive scores against state-of-the proprietary content writer tools while evaluated by LLM-as-a-judge approach in terms of content generation, context retrieval and citations. The evaluation scores were generated and evaluated in multiple runs by sensibly selected generator and evaluator model sets to avoid judgment bias as much as possible.

**Discussion:**

Discourse2Draft serves the need of an open-source draft writer maintaining reference authenticity, full control on outline management and content reproducibility. By coupling LLM-agnostic evidence-based content generation with traceable knowledge retrieval, Discourse2Draft streamlines the journey from informal textual query to publication-quality writing while keeping scientific rigor and author oversight at the forefront.

## 1 Introduction

The rapid proliferation of large-language models (LLMs) has catalyzed a new generation of “deep research” platforms that promise to accelerate literature discovery, hypothesis generation, and scientific writing. Tools such as Semantic Scholar's TLDR feature (Cachola et al., 2020), Elicit's question-answering workflow (Elicit, 2023), Research Rabbit's citation-network visualizations (ResearchRabbit, 2025), and Scholarcy's automated paper digests (Scholarcy, 2026) exemplify the growing ecosystem of systems that summarize findings, surface related work, or extract structured information from vast corpora. More recently, retrieval-augmented generation (RAG) frameworks—including GitHub Copilot for code and Scite's Smart Citation Generator (Nicholson et al., 2021)—have blended local knowledge bases with generative text to create context-aware outputs that more closely mimic domain expertise. Despite these advances, the majority of existing solutions fail to provide a consistent structured outline according to the user's intentions or authentic references to support the content. Many of these tools focus on gathering basic literature information (title and abstract) on a topic, often powered by a simple internet search or a limited number of document-summarization tools supporting narrow tasks. Few tools enable researchers to generate full, publication-grade manuscripts with a structured user-provided outline while preserving traceability to source material and providing explicit measures of factual certainty through authentic citation of peer-reviewed journals in a user-intended format.

Across the open-source ecosystem, AI-assisted scholarly writing tools span full-draft generation, literature-review automation, evidence-grounded retrieval, citation verification, and research knowledge-base construction. *Literature Review Agent* emphasizes auditable review writing through retrieved literature, user-approved outlines, local full-text evidence, evidence cards, and deterministic references (Littlelelephant, 2026). *OpenDraft* targets autonomous long-form academic drafting with multi-agent research, structured writing, citation filtering, and PDF/DOCX/LaTeX export (De Ponte, 2026). *STORM* and *Co-STORM* focus on topic exploration, outline synthesis, retrieval-grounded article generation, collaborative discourse, and mind-map-supported knowledge curation (Shao et al., 2024). *LitLLM* is narrower but relevant for RAG-supported literature review and related-work generation from a user idea, abstract, or writing plan (Agarwal et al., 2024). *ReLit* similarly supports citation-backed related-work generation using search, retrieval, filtering, summarization, and writing agents (Smith and Szatan, 2025). *IterSurvey/AutoSurveyV2* explores iterative academic survey generation through outline construction, paper cards, section drafting, and refinement loops (Zhang et al., 2025). *SurveyX* provides automated survey generation from titles, keywords, and prepared references, with a local open-source version oriented around user-supplied “Markdown” evidence (Liang et al., 2025). *InteractiveSurvey* adds a more interactive survey-generation workflow, allowing user refinement of paper filtering, outline structure, and generated survey content (Wen et al., 2025). *PaperQA2* is less of a writer and more of a scientific-document RAG engine, but it is important because of its stronger citation-grounded question answering over papers and local files (Skarlinski et al., 2024). *PubMedRAG* is especially relevant for biomedical workflows because it combines PubMed retrieval, semantic chunking, vector search, and citation-numbered responses (Liu, 2025). *Perspicacité-AI* focuses on local-first scientific knowledge bases, literature ingestion, provenance-aware RAG, DOI/BibTeX handling, and citation graph expansion (HolobiomicsLab, 2026). *Thoth* is the strongest citation-authentication comparator because it performs per-claim citation verification and labels cited claims as supported, unsupported, or unclear (Hobeishy, 2026). Together, these tools demonstrate that the open-source space is rich in retrieval, drafting, and citation support, but comparatively fewer tools combine strict user-controlled outline slots, editable manuscript workspace behavior, resumable section-level generation, reference-document upload, literature search, paragraph-level regeneration, and multi-format scholarly export in one application.

To address these needs, we developed *Discourse2Draft*, an open-source, AI-powered manuscript drafting system that supports structured, evidence-grounded writing while preserving user control over document organization, source use, and section-level revision. To evaluate the position of *Discourse2Draft* within the broader landscape of open-source AI writing tools, we selected four representative systems for comparison: *Literature Review Agent*, which emphasizes auditable literature-review generation (Littlelelephant, 2026); *OpenDraft*, which focuses on autonomous long-form academic drafting (De Ponte, 2026); *STORM/Co-STORM*, which supports knowledge-curation-based article generation through both autonomous and interactive workflows (Shao et al., 2024); and *Thoth*, which provides a systematic-review-oriented workflow with strong citation-audit features (Hobeishy, 2026). These tools were selected because they collectively cover the major design directions in current AI-assisted scholarly writing systems, including autonomous drafting, interactive knowledge curation, evidence-based review generation, and citation verification. The current version of *Discourse2Draft* offers greater flexibility as a human-editable structured drafting workspace than the majority of the reviewed systems, although tools like *Literature Review Agent* may offer a better architecture for evidence mapping during literature extraction. We have provided a more detailed comparison among different aspects of *Discourse2Draft* and the four tools in Supplementary Section 1.1 in Supplementary data.

In its current version, *Discourse2Draft* offers an open-source, end-to-end, query-to-manuscript pipeline that is simultaneously transparent, customizable, and model-agnostic. It provides full control over the outline of a manuscript (or any structured document) and appropriate citations to authentic publications downloaded from a comprehensive and trusted literature database, *PubMed Central*. After ingesting a user-supplied query, the application uses AI to shape an outline for the manuscript. It then writes each section of the outline using relevant information from user-provided documents, literature from *PubMed*, or both as AI context using a RAG approach. *Discourse2Draft* also provides granular controls such as domain, writing style, and size of the written document that should allow authors to modulate both the breadth and depth of the output. By coupling controls on both structure and content of a document with authentic references, *Discourse2Draft* empowers scientists to tailor AI-generated manuscripts to diverse journal formats, grant applications, or internal reports without sacrificing rigor or authorial oversight. *Discourse2Draft* also provides manageability by allowing users to regenerate and rephrase paragraphs with granular instructions and download the generated content and associated references in widely used editable document formats.

## 2 Methods

### 2.1 Installation

*Discourse2Draft* code can be downloaded from GitHub (https://github.com/amlantalukder/Discourse2Draft/releases/tag/v0.1.0). The repository contains a “README.md” file with step-by-step installation instructions. The user needs to configure the app to run on-premises. The mandatory configurations include the AI settings, content generation settings, RAG database, and main database settings. The user can learn more about these configurations in the “README.md” file.

### 2.2 User account and settings

*Discourse2Draft* can be accessed without creating an account, but a user account is helpful for storing the generated and uploaded documents and associated settings. The default LLM model name, temperature of the LLM (controls the creativity of LLM output generation), and instructions for the LLM can be set during installation, all of which can be changed by the user in the “Settings” panel.

### 2.3 User inputs

#### 2.3.1 Query and outline

*Discourse2Draft* accepts a user query (e.g., “Write a review article on Quantum Computing.”) and a structured outline as input. The user can choose to provide the outline or to generate one using an LLM based on the provided query. *Discourse2Draft* strictly follows the user-validated outline during content generation and allows the user full control over the generated content. The user can make changes to the generated outline using an interactive user interface (UI).

Query: The user can provide a query and upload a reference file (if necessary). Next, the user can click the “Create outline” button, which will generate a structured outline from the query, considering the reference file (if provided).

Structured Outline: The user can upload an outline in “Markdown (.md)” or “Microsoft Word (.docx)” format, choose an outline from the pre-uploaded or sample outline templates, or generate an outline using AI. Each of these steps generates a structured outline that follows a set of guidelines (provided in the next paragraph). The user can also choose to write the outline directly in the text area following the guidelines. The guidelines were designed to help the AI model parse the structured outline smoothly and identify places where it needs to generate content. They also help the AI model extract the pre-written summary of the content generated thus far, which is useful for maintaining continuity in the next generated content.

*Structured Outline Guidelines:* The structured outline must maintain the section hierarchy in standard “Markdown” format, where each hierarchical-level header is preceded by several “#”s. One “#” denotes the topmost level (which is typically the title of the outline), and increasing the number of “#”s denotes subsequent levels in the hierarchy (e.g., a header preceded by two “#”s defines the second hierarchical level, such as “Abstract,” “Introduction,” etc.). An outline must begin with one “#” to define the topmost level. The position where the AI needs to write the content has to be specified with a “[−−content−−]” tag. The user can add additional instructions for content generation under each section (or sub-section) by enclosing the instructions within “[−−Instructions−−]” and “[/−−Instructions−−]” tags (Figure 1).

**Figure 1:**
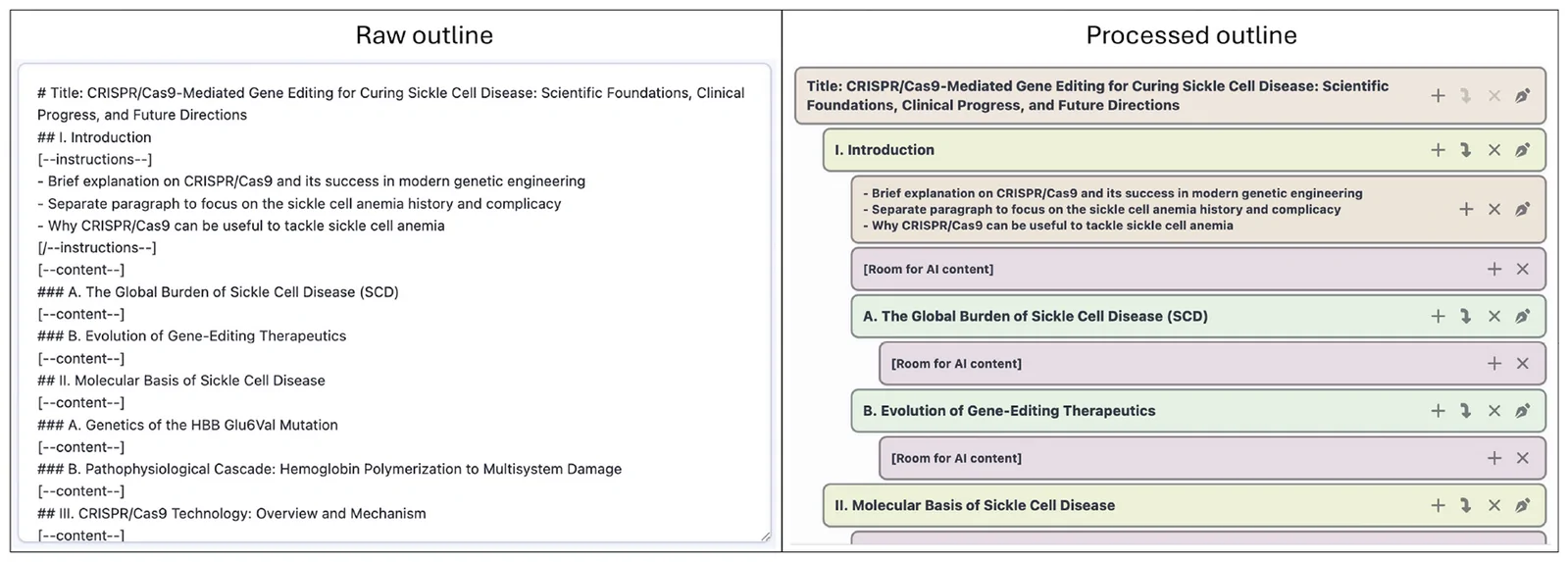
Raw outline input format for content creation. The **(Left)** panel displays an example of the structured outline with the necessary syntax. The **(Right)** panel shows the hierarchy of how the outline is processed from the raw text shown in the **(Left)** panel.

#### 2.3.2 Document upload

*Discourse2Draft* can use user-uploaded documents to gather context during content generation. The documents can be uploaded as “text (.txt),” “Microsoft Word (.docx),” or “.pdf” formats from the user's machine via the “Upload documents” panel in the middle of the left sidebar of the UI. The uploaded documents can later be attached to a new file for content generation. The IDs of the uploaded documents are stored in a PostgreSQL relational database associated with the user account (if the user signed in) or the active session (if the user did not sign in).

The user can also upload outline templates before generating content on the “Upload outline templates” panel in the bottom-left corner of the UI. This will allow the user to select the uploaded template during content generation. The accepted formats for the uploaded template files are “Markdown (.md)” and “Microsoft Word (.docx).”

### 2.4 Content generation

#### 2.4.1 Outline processing

To write a document, *Discourse2Draft* either takes a structured outline from the user or creates one based on the user-provided query described in Section 2.3.1. It then breaks the outline into a hierarchical structure (Figure 1), validates it, and detects the first position in the outline where it needs to write (denoted by the “[−−content−−]” tag). It then gathers the header chain specified by “#”s of that position from the topmost level (title → section → subsection → sub-subsection, etc.) and the generated content so far to ensure relevance and consistency during content generation. It also gathers the instructions for the corresponding section, if any are provided within “[−−Instructions−−]” and “[/−−Instructions−−]” tags. It then detects if the outline has an “Abstract” section or a section with a similar header, e.g., “Summary,” “Synopsis,” “Outline,” etc. *Discourse2Draft* uses an LLM to detect the presence of these fields. If the writable position is part of an “Abstract” section, the system skips this position and writes the Abstract at the end and moves on to the next writable position. To write in a position, *Discourse2Draft* passes the corresponding section header chain and the summary of the previous content to a suitable AI workflow (described later in this section). The AI workflow generates the new content and a summary of the document content up to the current position, which can be used to generate the next content (Figure 2).

**Figure 2:**
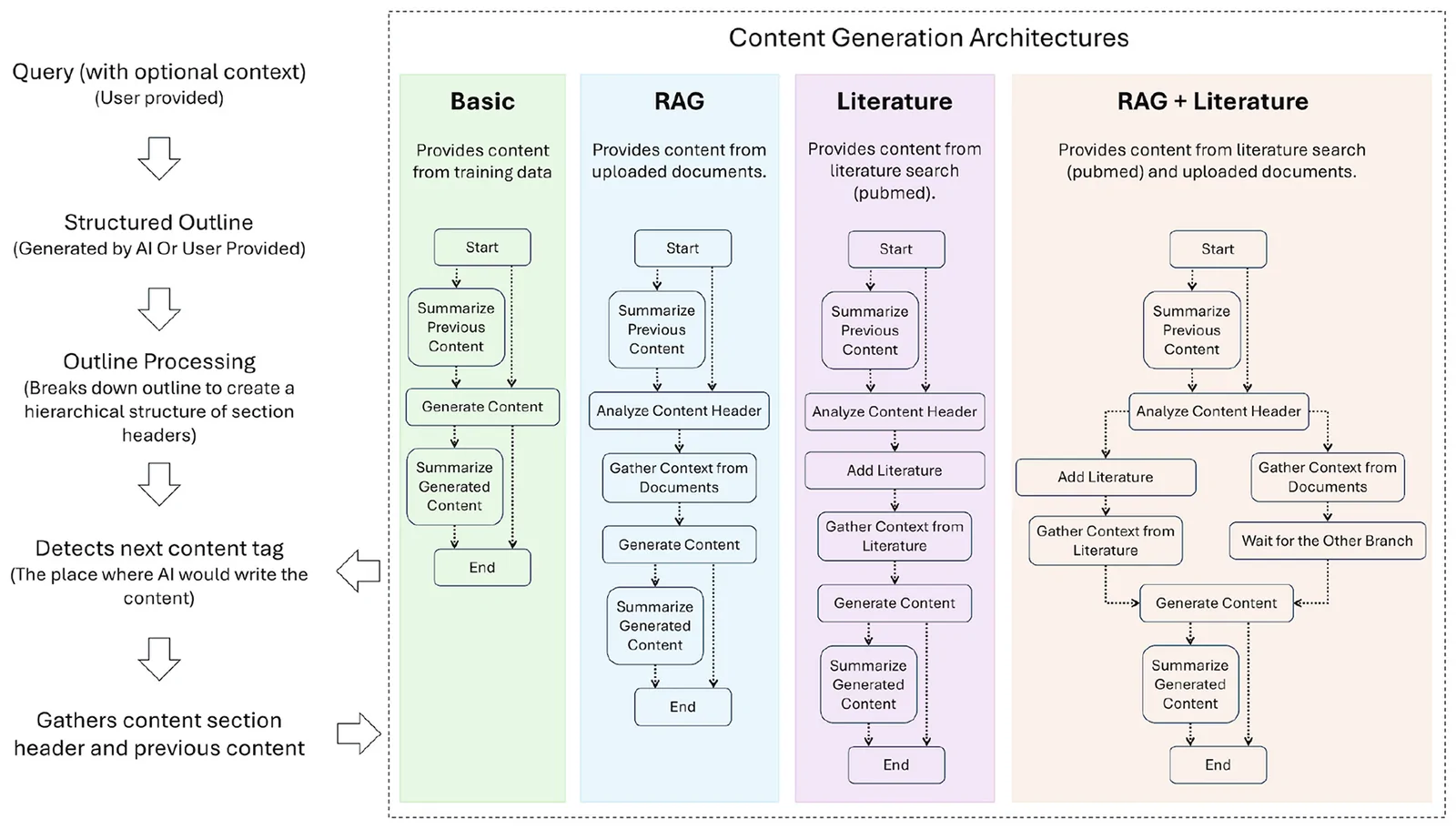
Behind-the-scenes user input processing steps and different AI workflows.

#### 2.4.2 AI workflows for content generation

*Discourse2Draft* follows different AI workflows based on user needs (Figure 2). Each workflow uses three inputs: the section header chain of the current position, the summary of the previously written content, and user-provided instructions for the given section extracted from the “[−−Instructions−−]” and “[/−−Instructions−−]” tags. Each workflow generates two outputs: the AI-generated content and the summary of the overall content (created by combining the generated content with the previous content summary). At the beginning of an AI workflow, *Discourse2Draft* summarizes the previously written content as an internal step if it contains more than 500 tokens to ensure that the number of context tokens does not exceed the limit of the underlying LLM. This step is usually redundant, and the next section already considers the summarized version of the previously written content; however, if the AI fails to summarize the content in the last step, this step helps to verify that the number of tokens remains within the threshold.

Basic: This workflow generates content from the pretrained knowledge of the associated LLM based on the headers and previously written content summary. This is the default workflow.

RAG: This workflow considers context from user-provided documents. When a user-provided document is attached to this workflow, the document is first divided into text chunks of 1,000 characters, with an overlap of 200 characters. Then, each chunk of text is converted into an embedding vector (called “vector document”) and saved into a vector database table (called “collection”), which we refer to here as the *document collection*. To generate content for a file with attached user documents, *Discourse2Draft* follows the “RAG” workflow, where the underlying LLM analyzes the previous content summary and current section headers to generate 10 semantically independent keyphrases relevant to the topic. Then, each keyphrase is searched for in the vector database collection to find 5 semantically similar “vector documents.” The keyphrases and the retrieved texts from the “vector documents” are combined and passed to the associated LLM as context. Finally, the LLM uses the provided context together with its pretrained knowledge to generate the final content.

Literature: Apart from the user-uploaded documents, *Discourse2Draft* can also gather the full text of a publication on the topic from an online literature database. This feature is enabled by selecting the “Literature Search” option at the top of the content panel (Figure 3). After that, *Discourse2Draft* follows similar steps to those in the “RAG” workflow, but it replaces the vector database search with a literature search. Similar to the “RAG” workflow, the system starts by analyzing the previous content summary and the current section header to generate 5 keyphrases. *Discourse2Draft* then uses each keyphrase to build a query string for the online literature database and uses the query string to extract the full text of the 2 most relevant articles. In this version of *Discourse2Draft*, we only use the open-access *PubMed Central* database as the literature database (Roberts, 2001) and extract the top 2 articles matching the keyphrase across “All fields” of the article using PubMed's default search algorithm. From each extracted piece of literature, *Discourse2Draft* uses the first 20,000 characters from each piece of literature. The extracted content of the literature is then processed in the same way as the “RAG” workflow and saved in the vector database collection, which we refer to here as the *literature collection*. The steps for content generation using these saved embeddings are the same as those in the “RAG” workflow.

**Figure 3:**
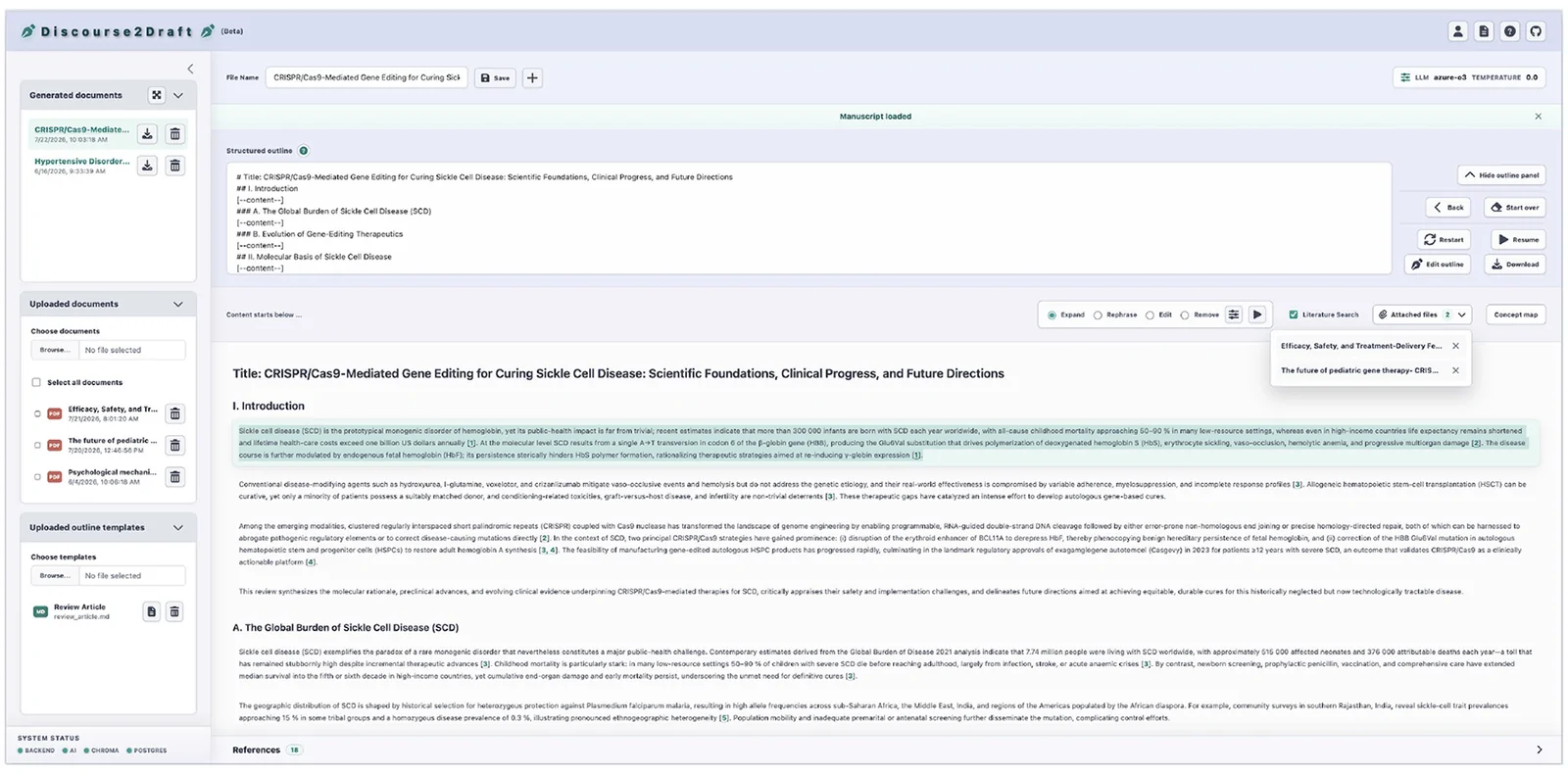
Content generation window of the app. The **(Top left)** and **(Bottom left)** panels show the generated and user-uploaded files. The **(Top right)** panel contains the outline. The **(Bottom right)** panel contains the generated content.

RAG + Literature: This workflow, as the name suggests, merges both “RAG” and “Literature” workflows. Each keyphrase, generated after analyzing the previous content and the current section headers, is used for searching both the *document collection* and the *literature collection* of the vector database to retrieve semantically similar text chunks from both sources. The text chunks are then passed to the associated LLM as context. In this way, the LLM considers the provided context from both uploaded documents and relevant literature retrieved through the literature search with its training data to generate the final content.

#### 2.4.3 Other AI workflows

In addition to content generation, *Discourse2Draft* uses AI workflows in outline generation and formatting and for abstract detection and writing. These workflows are simpler than the content generation workflows and are driven mainly by prompt engineering. The outline generation workflow is used when the user provides a text-based query as input, with or without a reference file. If a reference file is provided with the query, the outline generation workflow iteratively summarizes the content from the reference file and extracts a gist that fits within the associated LLM context token limit. When the user provides the structured outline directly as input, the outline formatter workflow is used to correct the outline, if needed. The abstract detection workflow is applied to determine whether the outline has an “Abstract” or a section with a similar header, e.g., “Summary,” “Synopsis,” “Outline,” etc. If there is an abstract, *Discourse2Draft* writes it using the abstract writer workflow after the entire document is written.

#### 2.4.4 Referencing

When the “RAG,” “Literature,” or “RAG + Literature” workflows are used, *Discourse2Draft* cites the generated text with references from the user-provided documents or online literature. A list of references to the cited documents and literature is shown in American Psychological Association (APA) format at the end of the document under the “References” section (Figure 3). It maintains the accuracy and order of every reference that is cited in the generated document.

When an uploaded document is attached, or the literature database is searched, a record with the metadata for the document/literature is created in *Discourse2Draft*'s relational database, and the full text of the document/literature is saved in chunks in *Discourse2Draft*'s vector database. In the above three workflows (as described in Section 2.4.2), the semantically relevant text chunks retrieved from the vector database are used as context for an LLM to generate the final content. The text chunks from different documents and literature sources, tagged with the corresponding record identifier (ID) of the document/literature from the relational database, are provided to the LLM as context. The LLM is instructed to cite the ID of a text chunk at the end of every group of sentences it generates using that text chunk. The cited ID is then programmatically tracked to retrieve the record with metadata for the document/literature from the relational database, which is then processed to create a citation at the specified position of the generated content and add the corresponding reference in the “References” section.

### 2.5 Modification of content

*Discourse2Draft* provides features that allow the user to modify each paragraph of the content after content generation. The user can expand or rephrase a paragraph with AI using specific, user-defined instructions. The user can also edit a paragraph manually (edit mode opens when the user either selects the “Edit” action or double-clicks the paragraph) or completely remove it from the content. The controls for regeneration are shown on the content bar when a paragraph is selected for modification (Figure 3).

### 2.6 Concept map

Each document is associated with a concept map, which is a tree of keyphrases explored by the AI during content generation under a section. This map can help the user obtain an overview of the document without reading it. It can also help the user validate the reasoning of the AI model during content generation (Supplementary Figure S2). The concept map can be accessed via the “Concept Map” button at the top of the content panel.

### 2.7 Custom instructions for AI

*Discourse2Draft* prioritizes following a set of regulations that are built into the system prompt. The user can add additional instructions in the settings menu that apply to the files generated under the user profile. As mentioned earlier, the user can also add instructions inside the structured outline within “[−−Instructions−−]” and “[/−−Instructions−−]” tags. These instructions are section-specific and are appended to the instructions provided in the user profile settings. In addition to these instructions, the user can also add instructions to regenerate a paragraph of a file's content. These instructions are appended at the end of the other user instructions in the LLM prompts.

### 2.8 Outputs

*Discourse2Draft* provides the ability to download the generated document in Markdown (.md), Microsoft Word (.docx), LaTeX (.tex), and PDF (.pdf) formats. Markdown, Microsoft Word, and PDF-formatted files contain the references inside the file. For the LaTeX-formatted file, the bibliography of the associated references can be downloaded separately in BibTeX format. The download options are shown on the right side of the outline panel in the generated documents panel and in its expanded view of the generated-document panel, which can be opened by clicking the expand button in the top-right corner of the panel (Figure 3). The concept map of a document can be downloaded in JSON format.

## 3 Evaluation

We evaluated *Discourse2Draft* by comparing it with two state-of-the-art AI tools for deep research: *OpenAI deep research* (OpenAI, 2025) and *Manus AI* (Shen et al., 2025) (Lite version), using the LLM-as-a-judge approach. As documents to generate, we selected three topics from the biomedical domain: “CRISPR-based therapeutic genome editing for inherited blood disorders,” “Phthalates Toxicity,” and “Health Impacts of Per- and Polyfluoroalkyl Substances (PFASs): A Comprehensive Review.” Each of these topics is well-researched in its respective domain, so each has many peer-reviewed articles in *PubMed Central*. We generated content about the three topics with the same prompt across all three tools. Each prompt contained an outline with a defined section hierarchy so that the tools can maintain the same structure and thus be compared with *Discourse2Draft*. For *Discourse2Draft*, we used the “Literature” workflow with the following generator and evaluator models,

- *Generator:* GPT 5.6 Terra; *Evaluators:* Gemini 3.5 Flash, Claude Haiku 4.5
- *Generator:* Gemini 3.1 Pro; *Evaluators:* GPT 5.6 Luna, Claude Haiku 4.5
- *Generator:* Claude Sonnet 4.6; *Evaluators:* GPT 5.6 Luna, Gemini-3.5 Flash

We chose LLMs from three leading LLM providers: OpenAI, Google, and Anthropic. For the generator models, we chose the models selected for a balance of reasoning capability, speed, and reliability. To evaluate a generator model from a provider, we chose a relatively fast model from the other two providers to minimize potential training biases. The generator models were run three times to generate the content on the three aforementioned topics. The evaluator models were used to score content generation, context retrieval, and citation quality of the “Literature” workflow.

Evaluation of “Content generation”: The content generated by a generator model on the three topics was evaluated by the two assigned evaluator models mentioned above. The evaluator models scored the content of each section of the three documents between 0 and 100 based on the following criteria:

*Relevance:* Is the content relevant to the provided section header and the previously written content summary (if provided)?

*Continuity:* Is the writing style of the content consistent with the previously written content summary? If the previously written content summary is not provided, the highest score (100) is assigned. This criterion is not used to score “References.”

*Non-repetitiveness:* Is the content devoid of repetitiveness compared with the previously written content summary? The highest score (100) is given when a previously written content summary is not provided.

*Specificity:* How precisely does the content explain the section?

Evaluation of “Context retrieval”: As mentioned in the Section 2.4.2, the “Literature” workflow creates 5 keyphrases, each of which is later used to retrieve context chunks from the vector database. The evaluator models were used to score both the “keyphrase generation” and “context retrieval” steps separately on each section of the documents generated on the three topics. Each score was set between 0 and 100 based on the following criteria:

Keyphrase generation:

*Relevance:* Are the keyphrases relevant to the provided section header and the previously written content summary (if provided)?

*Completeness:* Are the keyphrases sufficient to write the section?

*Specificity:* Can the keyphrases precisely support the content of the section based on the previously written content (if provided)?

Context retrieval:

*Relevance:* Is the retrieved context relevant to the provided section header and the previously written content summary (if provided)?

*Specificity:* Is the retrieved context able to precisely explain the section based on the previously written content summary (if provided)?

Evaluation of “Citation quality”: To evaluate the appropriateness of a citation, we separated and cross-referenced the sentences cited by a set of literature IDs and the corresponding context chunks retrieved from the vector database. For each literature ID, we then used the evaluators to score the context chunks from the literature ID against the sentences cited with the literature ID for relevance between 0 and 100.

*Relevance:* Is the retrieved literature context relevant to the generated content cited with the literature ID?

*Specificity:* Does the retrieved literature context precisely explain the generated content cited with the literature ID?

The above criteria were selected to evaluate the quality of AI-generated content. We chose these criteria because, unlike many other criteria such as tone and originality, they are easy for an LLM to evaluate using a score, which may reduce the risk of hallucinations during evaluation. For “content generation” evaluation, we have provided the LLM evaluation results on each criterion and compared the scores of three *Discourse2Draft* runs with *OpenAI deep research* and *Manus AI*. Each *Discourse2Draft* run shows a score bar with the mean and range of six values (three generator models each evaluated by two evaluator models), while *OpenAI deep research* and *Manus AI* scores show the mean and range of three values (the default model of the tool evaluated by three evaluator models) (Figure 4). Regarding the evaluation on “keyphrase generation,” “context retrieval,” and “citations,” we presented results from three *Discourse2Draft* runs, where each run shows a score bar with the mean and range of six values (three generator models each evaluated by two evaluator models) (Figures 5–7).

**Figure 4:**
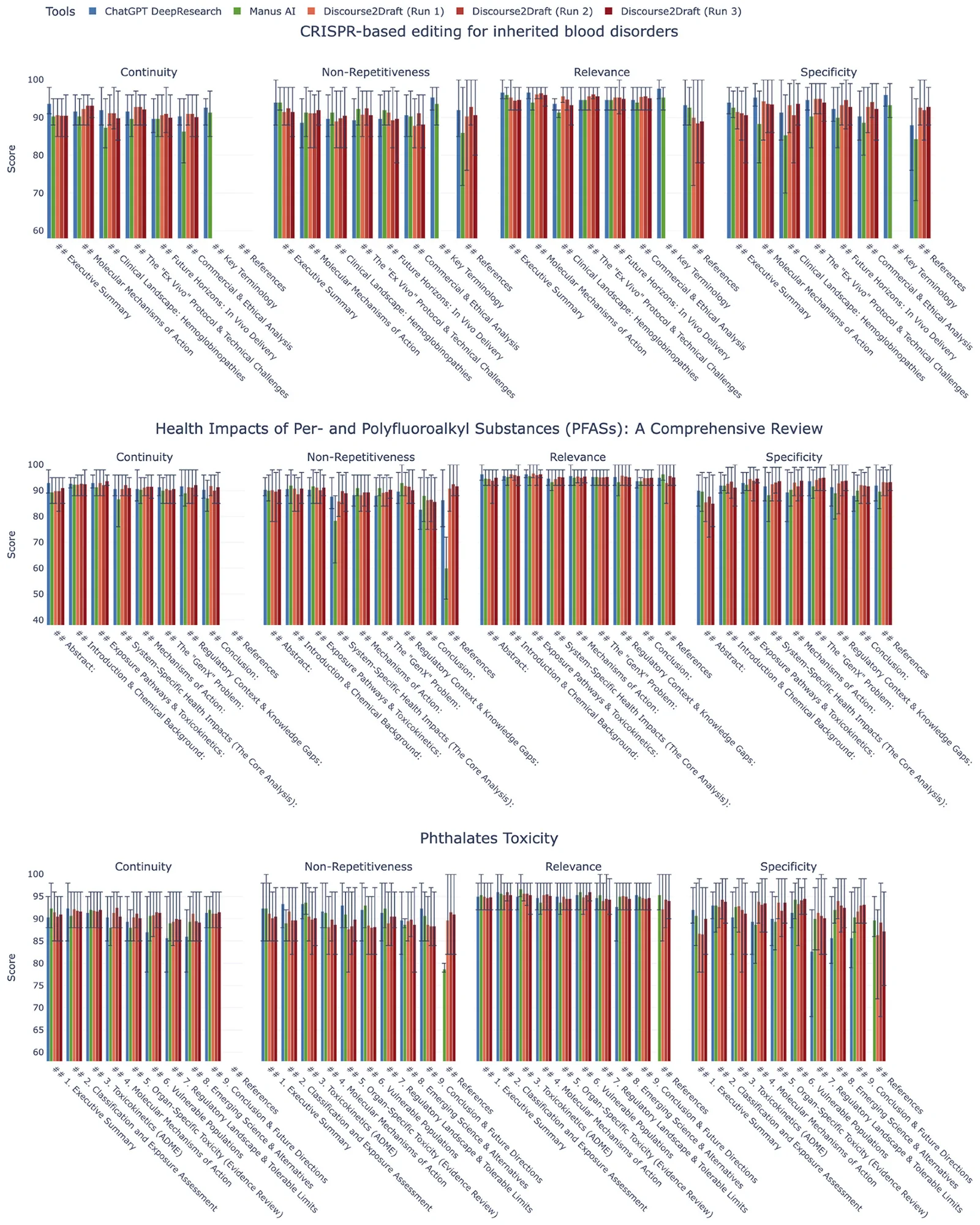
Evaluation scores on content generation. Each score bar shows the mean and range of six values (three generator models each evaluated by two evaluator models) for one Discourse2Draft run and three values for *OpenAI deep research* and *Manus AI* (the default model of the tool evaluated by three evaluator models).

**Figure 5:**
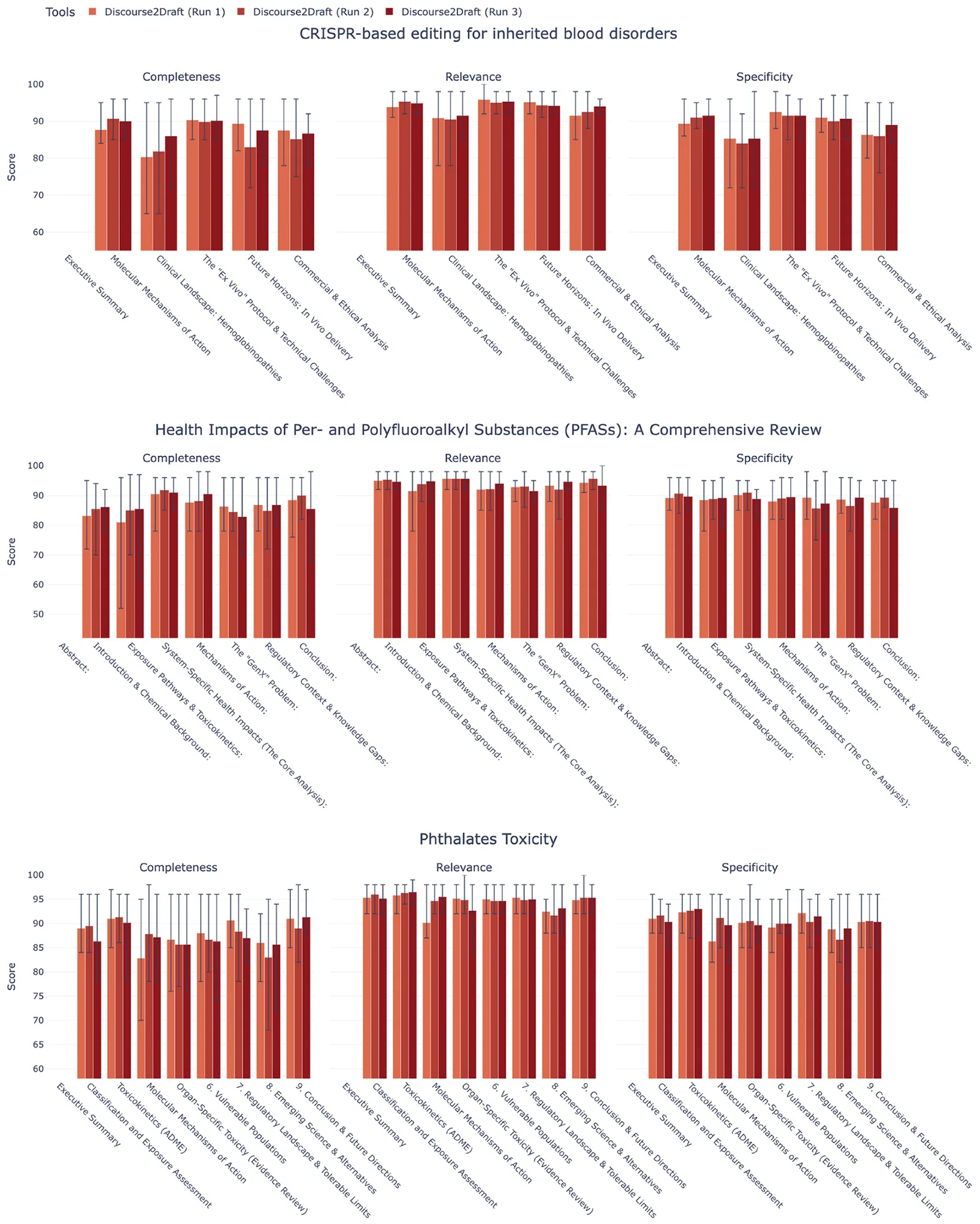
Evaluation scores on keyphrase generation. Each score bar shows the mean and range of six values (three generator models each evaluated by two evaluator models) for one *Discourse2Draft* run.

**Figure 6:**
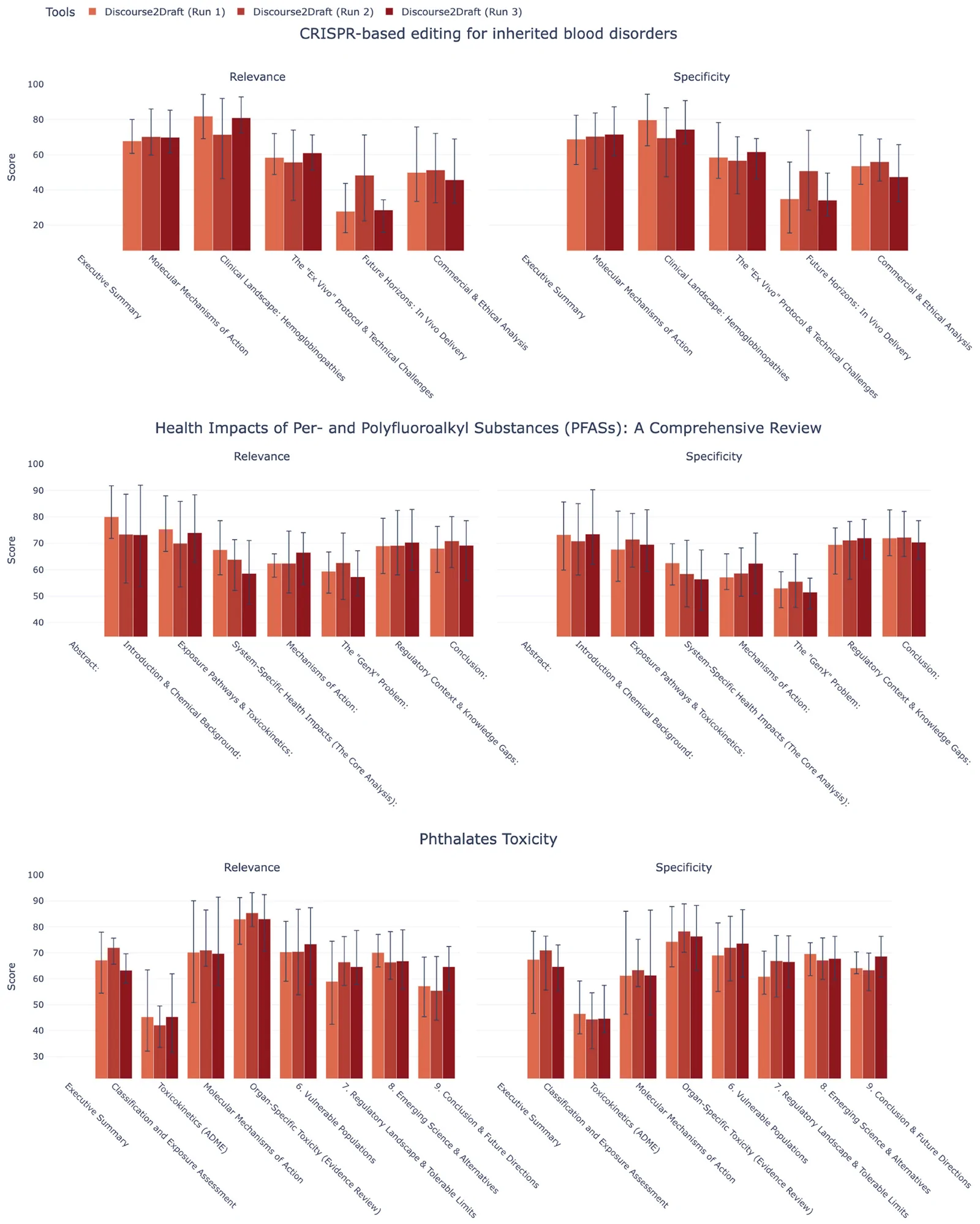
Evaluation scores on context retrieval. Each score bar shows the mean and range of six values (three generator models each evaluated by two evaluator models) for one *Discourse2Draft* run.

**Figure 7:**
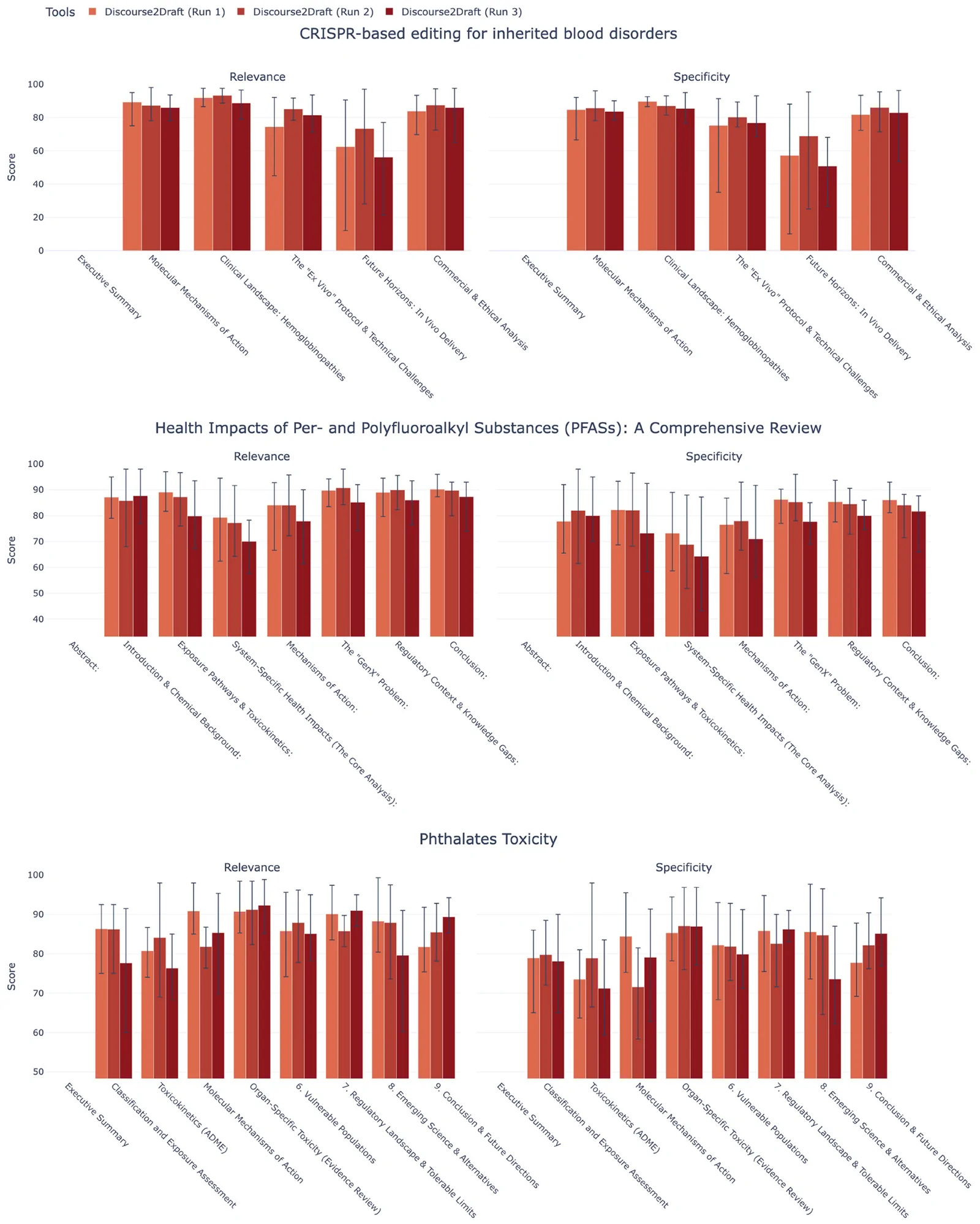
Evaluation scores on citations. Each score bar shows the mean and range of six values (three generator models, each evaluated by two evaluator models) for one *Discourse2Draft* run.

Regarding content generation, *Discourse2Draft* generally maintained close alignment with section headers and thematic expectations as reflected in the *relevance* scores across the evaluated documents. This indicates that *Discourse2Draft* effectively interpreted structured prompts and produced content that adhered to the intended topical scope, a critical requirement for scientific and review-style writing. *Discourse2Draft* also demonstrated solid performance on *continuity* scores, despite using a summary of the previous content controlled by a maximum-token threshold (500). Its outputs tended to preserve a coherent narrative flow and stylistic consistency across sections, suggesting that it effectively leverages contextual inputs. This is especially important in multi-section biomedical documents, where logical progression and conceptual linkage between sections enhance readability and scholarly quality. Although its performance was not as strong as that for the previous two criteria, *non-repetitiveness, Discourse2Draft* appeared capable of avoiding redundant phrasing and content overlap with previously generated sections. This contributes to more concise and information-dense writing, which is desirable in technical domains where redundancy can obscure key insights.

To highlight some of the weaknesses in content generation, *Discourse2Draft* exhibited limitations in specificity, particularly in complex biomedical contexts. Instances of overgeneralization and occasional hallucination-like behavior suggest that the model may struggle to ground its outputs in precise, verifiable scientific detail. This is a critical shortcoming, as specificity and factual accuracy are paramount in biomedical writing. Another observed weakness is inconsistent performance across document sections and topics. Although *Discourse2Draft* performs well in structurally straightforward sections (e.g., introductions or summaries), its performance may degrade in more technically demanding sections such as mechanistic explanations or regulatory analyses. This variability suggests that the model's internal knowledge integration and reasoning depth may not be uniformly robust across domains. These shortcomings can be addressed by providing additional information to the AI workflow. This can be done by increasing the number of tokens in the previous content summary, the number of literature sources, and the content size of each manuscript, represented by 500, 2, and 20,000, respectively, in the default settings. In addition, *Discourse2Draft* used a simplistic workflow to download literature documents from only one source (*PubMed Central*), while the other two proprietary tools use networks of agents (OpenAI, 2025; Shen et al., 2025) to perform deep research using a wide variety of sources on the internet. Using open-source *PubMed Central* as the only literature source in the content generation may also have limited access to appropriate sources that could have better supported the AI model to generate high-quality content. Despite these limitations, *Discourse2Draft* was able to maintain a competitive score on all the criteria above and consistently higher scores for the “References.”

The generated keyphrases were mostly rated highly by the evaluators in terms of completeness, relevance, and specificity, which demonstrates that the underlying generator models were appropriately aligned with the requirements in understanding what was needed to write a section given the section header and the previously written content summary. But the poor scores (below 50 out of 100) during context retrieval under some sections, e.g., “Future Horizons: *In Vivo* Delivery” on the topic “CRISPR-based editing...” or “Toxicokinetics (absorption, distribution, metabolism, and excretion [ADME])” on the topic “Phthalates Toxicity,” demonstrate that the retrieved content was not always used by the model to write a section. This is because the current literature extraction method considers only the top 2 literature sources (by default) for a section based on the default search strategy of the online literature database. For the poorly scored sections, the content of the extracted literature might not be considered ideal by the generator model to write those sections, and the generator model eventually relies on its pretrained knowledge for additional context. A stronger literature extraction strategy would involve careful screening and content extraction using evidence mapping, which we plan to provide in the next version of the tool. Citation quality was high in the majority of cases because each citation was cross-referenced against articles in the database before inclusion, reducing the risk of AI-fabricated references. However, the final content occasionally deviated from the cited literature, indicating that content-level hallucinations may still occur during content generation.

The evaluation process itself had limitations. Both content generation and evaluation processes were somewhat stochastic, as reflected in the range of scores across three runs of *Discourse2Draft*. Adding a human-in-the-loop strategy alongside the current LLM-as-a-judge approach would greatly strengthen the process, but we had to omit it because of limited subject-matter expert resources and the experts' limited availability. The selected metrics may also not be comprehensive, as selecting appropriate metrics that can rank the AI-generated content objectively is still a non-trivial problem.

## 4 Discussion

We aimed to develop an open-source, AI-powered document generation tool that can efficiently and reliably assist researchers in generating drafts while balancing flexibility with comprehensive content control. *Discourse2Draft* can generate structured content as can numerous other tools available now; however, unlike the majority of these tools, it allows the user to maintain control over the structure of the content. *Discourse2Draft* users can define or upload outlines, provide reference documents, revise individual sections, and guide the drafting process through iterative instructions. This design supports a human-in-the-loop workflow in which AI assists with content generation while the user retains authority over organization, emphasis, and final interpretation. Content generation is fully automated and can run in the background, making it suitable for long-running tasks.

*Discourse2Draft* integrates retrieval-based citation support and cross-references inserted citations with retrieved articles, reducing the risk of fabricated references while preserving transparency in the evidence used for drafting. The references inserted into the generated content are validated against retrieved articles before inclusion. This process helps ensure that the cited references correspond to real retrieved articles rather than AI-fabricated sources. Although the references themselves are validated, the generated text may still occasionally diverge from the cited literature, indicating that a low level of hallucination at the content level can still occur during writing.

*Discourse2Draft* demonstrates a balanced profile in structural alignment, coherence, and non-redundancy, making it well-suited for generating organized draft documents. However, the current version does not yet support evidence mapping during literature extraction, which may limit specificity, depth of scientific reasoning, and suitability for high-stakes biomedical writing tasks. This limitation is especially important because each section is generated from a small number of retrieved articles by default, placing parts of the workflow in a low-resource evidence setting. Recent work on low-resource learning emphasizes that robust generalization with limited data or evidence requires careful sampling, optimization, transfer, feedback, and structure-aware modeling (Cao et al., 2025). Prior work on mentored learning frames model improvement as a guided process in which a learner benefits from external teaching signals, including in black-box settings where the teacher cannot directly modify the learner's internal parameters (Cao et al., 2024). This perspective is relevant to AI-assisted manuscript generation because document quality depends not only on the generative model but also on how evidence, structure, user feedback, and discourse-level instructions guide the model toward the target document. In addition to factual grounding under limited evidence, allowing content-based literature selection, evidence mapping, adaptive query expansion, and source reranking can strengthen domain-specific reasoning capabilities to better compete with leading deep research AI systems.

In future versions of *Discourse2Draft*, we will focus on literature selection with evidence maps to enhance factual grounding and domain-specific deep reasoning abilities. In addition, to allow more control over the generated content, users will be able to review, accept, or reject references that would then reshape the generated content. We used *PubMed Central*, a free full-text database, which primarily focuses on articles published in the biomedical domain. To allow diversity in domains, we plan to add more online databases that provide open access to full-text articles (e.g., Web of Science and Scopus). The default settings in the current version were configured to keep the underlying workflows simple while maintaining a balance between speed and accuracy. To maintain simplicity, this version does not support figures or illustrations; however, we plan to add this feature in a future version. Although a formal human expert review of the generated content was beyond the scope of the current release, formalizing the incorporation of expert review is a priority for future versions. Despite these limitations, the current version can generate drafts for a variety of structured academic documents such as manuscripts for research articles, literature reviews, research proposals, theses and dissertations, case studies, and systematic reviews. The codebase is available for users to download and install so that it can be extended and improved by the community as needed.

## Data Availability

The original contributions presented in the study are included in the article/Supplementary material, further inquiries can be directed to the corresponding author.
