# Supplementary material for: Discourse2Draft: an AI-powered structured document writing tool
Source: Front Artif Intell. 2026 Oct 27;9:1945655. doi: 10.3389/frai.2026.1945655 (PMC13753207; doi:10.3389/frai.2026.1945655)
Supplement: Supplementary file 1 [file Data_Sheet_1.zip › Supplementary_files/supplementary_data.pdf]

Discourse2Draft: An AI powered structured document writing tool

1 SUPPLEMENTARY INFORMATION

1.1 Comparison with available open-source tools

We ran an in-depth comparison between *Discourse2Draft* and four of the most relevant recent open-source AI content writing tools: *Literature Review Agent*, *OpenDraft*, *STORM/Co-STORM*, and *Thoth* littlelelephant (2026); De Ponte (2026); Shao et al. (2024); Hobeishy (2026). The strength of the current version of *Discourse2Draft* lies in human-editable scholarly drafting workspace, as it gives the user more practical control over the writing process through strict outline templates, uploaded reference documents, resumable section generation, paragraph-level regeneration or expansion, and export-oriented manuscript handling. This makes *Discourse2Draft* better suited for users who already know the structure of the document they want and need an interactive tool to iteratively shape a manuscript. *Literature Review Agent* is stronger for auditable, evidence-grounded review writing because it preserves intermediate artifacts, approved outlines, evidence cards, and resumable workflows, but it is less polished as a flexible web-based writing editor. *OpenDraft* is stronger for rapid autonomous long-form paper generation and export, but it gives the user less fine-grained control over individual sections and paragraphs. *STORM/Co-STORM* is stronger for broad topic exploration, multi-perspective research, and cited report generation, but it is less focused on manuscript editing, strict outline compliance, and publication-style export workflows. Thoth is the strongest comparator for authenticity because of its per-claim citation verification and systematic-review pipeline, while *Discourse2Draft* could improve by adding claim-level citation checking, citation-choice controls, stronger hallucination audits, and more transparent evidence-trace reports. We provided a feature-wise comparison among these tools highlighting their strengths and weaknesses to provide a better view of where *Discourse2Draft* could be useful (Fig S1). In a nutshell, *Discourse2Draft*'s advantage is flexible, user-guided structured drafting, while its main improvement opportunity is deeper citation authentication and auditability.

1.2 Concept map

During content writing, it is often useful to see how the AI model thinks while making connections with the provided context. *Discourse2Draft* provides a way to see the section-wise concept connections that were pivotal for the model to generate content for that section in the form of a tree of concept keyphrases, which we call "Concept map". This map can help the user get an overview of the document without reading it. It can also help the user validate the thoughts of the AI model during content generation (Fig. S2). The concept map can be accessed via the "Concept Map" button at the top of the content panel.

1.3 Technology Stack

Discourse2Draft was built using the Python programming language. The front-end of *Discourse2Draft* was developed using ReactJS/Vite. PostgreSQL was used for the relational database in the back-end. The LangChain and LangGraph packages were used to create and manage the AI workflows. Chroma was used as the vector database (Fig. S3).

1.4 Evaluation

We compared the performance of *Discourse2Draft* with state-of-the-art AI content writing with deep research tools: *OpenAI deep research* (OpenAI (2025)) and *Manus AI* (Shen et al. (2025)) (Lite version) by evaluating the quality of the generated content on three topics. We ran *Discourse2Draft* three times using multiple generator and evaluator model sets, shown below. We also evaluated the context extraction and citation quality for *Discourse2Draft* separately.

- *Generator:* GPT 5.6 Terra; *Evaluators:* Gemini 3.5 Flash, Claude Haiku 4.5
- *Generator:* Gemini 3.1 Pro; *Evaluators:* GPT 5.6 Luna, Claude Haiku 4.5
- *Generator:* Claude Sonnet 4.6; *Evaluators:* GPT 5.6 Luna, Gemini-3.5 Flash

The prompts, generated contents by OpenAI deep research, Manus AI and Discourse2Draft and the evaluation results (scores and reasons) by the evaluator models are provided as supplementary data in a separate directory (*supplementary_data*).

1.4.1 Generated content

The generated content by the three tools are provided under *generated_content/<tool_name>* directory. The *<tool_name>* directory name takes three values: *discourse2draft* (*Discourse2Draft*), *chatgpt_deepresearch* (OpenAI deep research) and *manus_ai* (Manus AI). Each directory contains the content in "Markdown (.md)" format on the three topics.

- CRISPR-based editing for inherited blood disorders.md
- Health Impacts of Per- and Polyfluoroalkyl Substances (PFASs): A Comprehensive Review.md
- Phthalates Toxicity.md

1.4.2 Prompts

We used the same prompts, each containing the outline of the corresponding topic, to make it easier to compare the generated contents by the three tools. The prompts are provided in "Markdown (.md)" format in the *prompts* directory.

1.4.3 Results

The results are provided in *results* directory. This directory contains evaluation results on content generation (*content_generation*), keyphrases generation (*keyphrases_generation*), context retrieval (*context_retrieval*) and citations (*citations*). Each of these directories contains the scores and reasons in "CSV (.csv)" format provided by the evaluator models.

- reasons
  - <evaluator_model>
    - CRISPR-based editing for inherited blood disorders.csv
    - Health Impacts of Per- and Polyfluoroalkyl Substances (PFASs): A Comprehensive Review.csv
    - Phthalates Toxicity.csv
- scores
  - <evaluator_model>
    - CRISPR-based editing for inherited blood disorders.csv
    - Health Impacts of Per- and Polyfluoroalkyl Substances (PFASs): A Comprehensive Review.csv

- Phthalates Toxicity.csv

The ".csv" files contain the section names of corresponding generated document as columns and a combined string "discourse2draft|<generator_model>|run_<run_index>|criterion" representing the generator model, run index and the evaluation criterion as rows. In case of the evaluation of keyphrases generation (*keyphrases_generation*), context retrieval (*context_retrieval*) and citations (*citations*), the score represents a mean score and the reason represents the concatenated reasons by "|" symbol for all canonical evaluations (e.g. evaluation for one keyphrase or one context chunk) under a row and column.

REFERENCES

[Dataset] De Ponte, F. (2026). OpenDraft: Ai research draft generator. `https://github.com/federicodeponte/opendraft`. Software repository

[Dataset] Hobeishy, A. (2026). Thoth: Agentic systematic literature reviews with per-claim citation verification. `https://github.com/ahmedEid1/thoth`. Software repository

[Dataset] littlelelephant (2026). Literature Review Agent: An auditable and resumable multi-agent workflow for scholarly retrieval and evidence-grounded literature review writing. `https://github.com/littlelelephant/literature-review-agent`. Software repository. Accessed 2026-09-03

[Dataset] OpenAI (2025). Introducing deep research. `https://openai.com/index/introducing-deep-research/`

Shao, Y., Jiang, Y., Kanell, T., Xu, P., Khattab, O., and Lam, M. (2024). Assisting in writing Wikipedia-like articles from scratch with large language models. In *Proceedings of the 2024 Conference of the North American Chapter of the Association for Computational Linguistics: Human Language Technologies* (Association for Computational Linguistics), 6252–6278. doi:10.18653/v1/2024.naacl-long.347

Shen, M., Li, Y., Chen, L., and Yang, Q. (2025). From mind to machine: The rise of manus ai as a fully autonomous digital agent. *arXiv preprint arXiv:2505.02024*

FIGURE CAPTIONS

**Criterion**	**Discourse2Draft**	**Literature Review Agent**	**OpenDraft**	**STORM / Co-STORM**	**Thoth**
Main focus	Flexible and fast structured drafting workspace	Auditable literature-review writing	Autonomous academic draft generation	Knowledge curation and cited article generation	Systematic review with citation audit
Follows outline	**High**: Follows outline by design	**High**: Outline approval and paper selection	**Low**: Mostly autonomous	**Medium**: Co-STORM supports steering	**High**: Required approval gates
Pause/Resume writing	**High**: Yes	**High**: Checkpoints/sessions	**Low**: Not clearly section-level	**Medium**: Partial stage reuse	**Medium**: Partial durable workflow
Paragraph regeneration	**High**: Expand, rephrase, edit, remove	**Low**: Not central	**Low**: Mainly whole-draft revision	**Low**: Not central	**Low**: Not central
Allows outline/templates upload	**High**: Strong	**High**: User Markdown outline	**Low**: Limited/unclear	**Medium**: Limited/partial	**Medium**: Review plan, not template driven
Allows reference document upload	**High**: PDFs/DOCX/text	**High**: Local PDFs/Markdown	**Medium**: Paper input for some utilities	**High**: Through VectorRM	**High**: Uploaded/hybrid workflow
Citation authenticity	**Medium**: Chooses right references from pre-extracted list. No evidence mapping.	**High**: Deterministic references from real evidence	**High**: DOI/source existence checks	**Medium**: Retrieval-grounded citations	**High**: Per-claim citation check
Hallucination controls	**Medium**: RAG, PubMed, citation processing, concept map	**High**: Evidence cards, audits, coverage analysis	**Medium**: DOI confirmation and relevance checks	**Medium**: Retrieval grounding; review still needed	**High**: Supported/unsupported/unclear audit
Flexibility in export formats	**High**: PDF, MarkDown, DOCX, LaTeX ZIP/BibTeX	**Medium**: Mainly MD/JSON artifacts	**High**: PDF, DOCX, LaTeX	**Low**: Article/report output	**Low**: Markdown/audit outputs
Background/autonomous run	**High**: Yes	**Medium**: Partial	**High**: Yes	**Medium**: STORM autonomous; Co-STORM interactive	**Medium**: Gates prevent unattended runs
User interface	**High**: Yes	**Low**: No polished web UI	**Medium**: Hosted app; OSS mainly engine	**Medium**: Live preview/demo light	**High**: Yes
Ease of setup	**High**: Easy set up. Multiple set up options.	**Medium**: Local setup required	**High**: Easy-medium	**Medium**: LLM/retrieval setup required	**Low**: Medium-hard full stack

Figure S1. Strengths and weaknesses of *Discourse2Draft* in comparison with the four most relevant open-source tools to date.

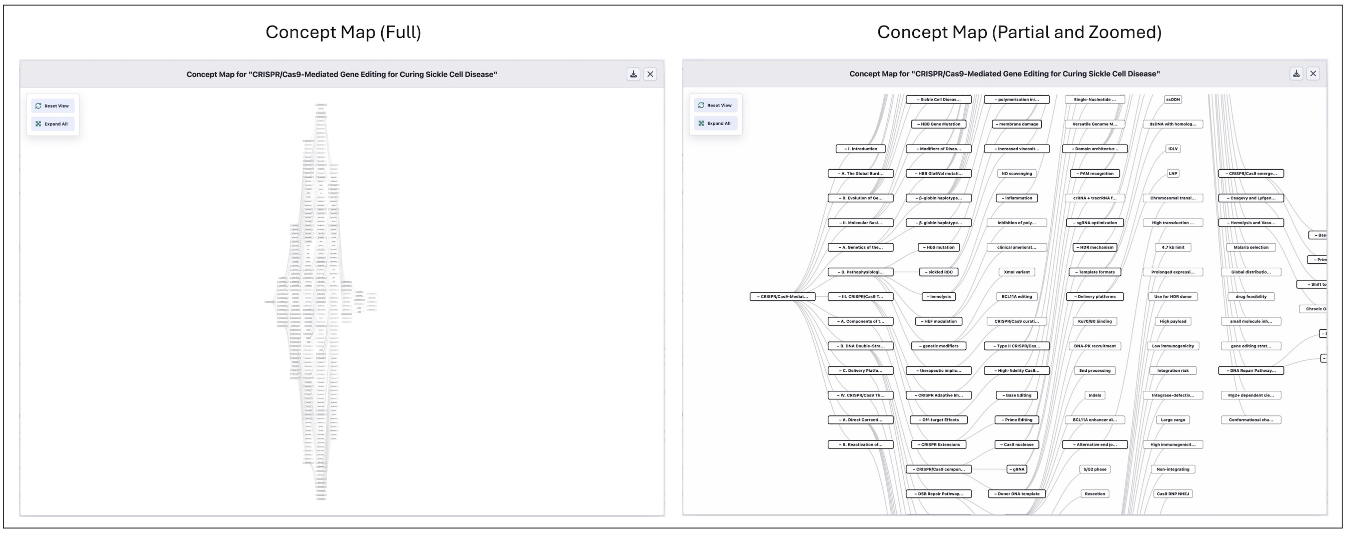

Figure S2. Concept map of a document generated by *Discourse2Draft*.

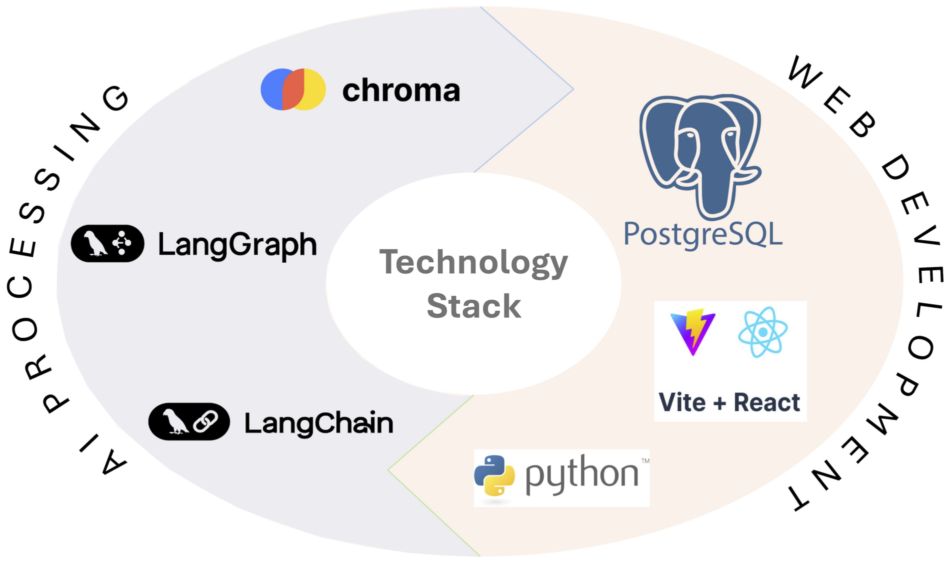

Figure S3. The technology stack used to develop the app.
